# Development of a Cognitive Impairment Discrimination Model through Acoustic Analysis of Speech Sounds

**DOI:** 10.1002/alz.088486

**Published:** 2025-01-09

**Authors:** Jin Yong Jeon, Haram Lee, Hyowon Yoon, Sungwon Roh, Donghyun Ahn

**Affiliations:** ^1^ Hanyang University, Seoul, Seoul Korea, Republic of (South)

## Abstract

**Background:**

This study investigates the impact of cognitive impairments on the acoustic characteristics of speech and explores the development of a non‐invasive AI model for the diagnosis of Mild Cognitive Impairment (MCI).

**Method:**

Specifically, using conversational speech data from MCI patients and healthy controls in the DementiaBank, the autocorrelation function (ACF) of the speech signal according to delay time was analyzed. Through such analysis, key acoustic indicators of the speech signal such as τ_1_, ϕ_1_, and τ_e_, were calculated, and these were used to employ the concept of pitch strength in the diagnosis of MCI. Additionally, by analyzing four instances of speeches given by former President Reagan at five‐year intervals, the study investigated the changes in speech that occur during the development of cognitive impairment.

**Result:**

The results indicated statistically significant differences in the vocal data of MCI patients compared to the healthy control group, suggesting that cognitive decline affects the periodicity and pitch characteristics of speech.

**Conclusion:**

However, findings from DementiaBank data, which involved responses to memory and cognitive‐function‐related queries in conversations between medical personnel and patients, were not observed in speech acoustic analysis data from simple reading tasks. This highlights the need for further experiments analyzing responses to semi‐structured questions, which involve cognitive brain activity, to confirm the utility of speech data in diagnosing patients with cognitive impairments.

